# Integrated Multiomics Analyses Reveal Molecular Insights into How Intermittent Fasting Ameliorates Obesity and Increases Fertility in Male Mice

**DOI:** 10.3390/nu17061029

**Published:** 2025-03-14

**Authors:** Shuyu Zhang, Tingting Lin, Yucheng Bao, Junsen She, Xuanqi Liu, Jiaxue Hu, Aibing Peng, Xinmei Liu, Hefeng Huang

**Affiliations:** 1 The International Peace Maternity and Child Health Hospital, Shanghai Key Laboratory of Embryo Original Disease, School of Medicine, Shanghai Jiao Tong University, Shanghai 200030, China; 2 Shanghai Key Laboratory of Reproduction and Development, Shanghai 200030, China; 3 Key Laboratory of Reproductive Genetics, Ministry of Education, Department of Reproductive Endocrinology, Women’s Hospital, Zhejiang University School of Medicine, Hangzhou 310058, China; 4 Ruijin Hospital, Shanghai Jiao Tong University School of Medicine, Shanghai 200003, China; 5 International Institutes of Medicine, The Fourth Affiliated Hospital, Zhejiang University School of Medicine, Yiwu 322000, China; 6 Department of Neurobiology and Department of Psychiatry of the Second Affiliated Hospital, Zhejiang University School of Medicine, Hangzhou 310058, China; 7 Obstetrics and Gynecology Hospital, Institute of Reproduction and Development, Fudan University, Shanghai 200030, China; 8 Research Units of Embryo Original Diseases, Chinese Academy of Medical Sciences, Shanghai 200030, China

**Keywords:** obesity, intermittent fasting, metabolic syndrome, male infertility, metabolomics, transcriptomics

## Abstract

**Background:** Intermittent fasting (IF) has been increasingly recognized for its potential to mitigate obesity and diabetes. However, it remains unclear whether IF can alleviate metabolic disorder-induced male infertility. The aim of this study was to investigate the potential of IF to improve fertility outcomes in obese mice. **Methods:** Eight-week-old C57BL/6J mice were fed a high-fat diet (HFD) for 24 weeks to induce obesity, followed by alternate-day fasting for 6 weeks. We assessed obesity-related metabolic changes and fertility issues postintervention. Comprehensive metabolomic and transcriptomic analyses of serum and testicular samples were used to identify significant metabolic pathway modifications attributable to IF. **Results:** IF effectively alleviated obesity-induced male infertility, demonstrating significant attenuation of body weight gain and restoration of testicular morphology. IF normalized hypogonadism-associated testosterone depletion and improved sperm parameters. Testis multi-omics integration revealed IF-mediated reprogramming of testicular purine metabolism, coupled with coordinated regulation of glycolipid metabolism and inflammatory-immune homeostasis. Reproductive competence was enhanced as evidenced by statistically elevated successful mating rates and embryonic developmental progression. Serum metabolomics further identified metabolites involved in amino acid metabolism, glycolipid metabolism, and inflammation (e.g., methionine, BCAA, glutathione, and spermidine) may serve as potential targets for treating obesity-related metabolic disorders. Additionally, multidimensional analysis highlighted the crucial role of allantoin in alleviating obesity and related reproductive dysfunction. **Conclusions:** IF not only resolves obesity-induced metabolic issues but also alleviates male infertility by regulating bioactive metabolites and gene expression linked to glycolipid metabolism, energy homeostasis, and immune responses in the testis. Our study provides a theoretical basis for IF as a clinical treatment for obesity-induced male infertility.

## 1. Introduction

Obesity is a complex, multifactorial disease whose global prevalence has more than tripled since 1980 [1]. Recent research in China indicates that obesity is more prevalent among men than women (18.2% vs. 9.4%), with the highest rates observed in men aged 35 to 39 years and in women aged 70 to 74 years [2]. Men are at increased risk of developing obesity during their reproductive years. In addition to the risks of cardiovascular diseases, diabetes and metabolic disorders, obesity is also a risk factor for male infertility [3]. Epidemiological studies have revealed a negative correlation between male obesity and various aspects of male fertility, including semen quality, sperm DNA integrity, and live birth rates through both natural and assisted conception [4,5]. Animal studies have revealed that obesity, particularly that induced by a high-fat diet (HFD), undermines male reproductive potential through disruptions in the hypothalamic-pituitary-gonadal (HPG) axis, hormonal regulation and increased oxidative stress, among other effects [6]. Notably, obesity-induced HPG axis dysfunction involves leptin resistance-mediated suppression of hypothalamic kisspeptin signaling, which impairs pulsatile gonadotropin-releasing hormone (GnRH) secretion. This suppresses pituitary synthesis of luteinizing hormone (LH) and follicle-stimulating hormone (FSH)—key drivers of testicular steroidogenesis [7]. Concurrently, Leydig cell dysfunction—manifested through downregulation of steroidogenic acute regulatory protein (StAR) and compromised testosterone synthesis—is exacerbated by adipose-derived aromatase converting testosterone to estradiol [8]. The consequent estradiol surge amplifies negative feedback on the HPG axis, while obesity-associated hyperinsulinemia reduces sex hormone-binding globulin (SHBG), thereby enhancing free testosterone availability for aromatization [9]. These hierarchical perturbations collectively culminate in hypogonadism and spermatogenic failure. Moreover, obesity can lead to the transmission of metabolic and neurodevelopmental disorders to offspring through genetic and epigenetic changes in sperm [10,11].

Given these challenges, there has been increasing interest in dietary modification as a strategy for obesity management [12,13,14]. Intermittent fasting (IF), which involves alternating periods of eating and fasting, encompasses three primary regimens: alternate-day fasting (ADF), the 5:2 diet, and time-restricted eating (TRE) [15]. IF has shown promise in enhancing metabolic health, reducing fat mass, improving glucose tolerance, and even extending lifespan [16,17,18]. Clinical studies have demonstrated significant benefits of IF in populations with obesity. Notably, a randomized controlled trial revealed that ADF combined with exercise effectively reduces body weight and low-density lipoprotein cholesterol (LDL-C) levels in cohorts with obesity [19]. Investigations of Ramadan fasting patterns further suggest this regimen may attenuate oxidative stress and enhance systemic metabolic regulation in nondiabetic individuals with obesity [20]. These clinical observations align with preclinical evidence showing IF effectively ameliorates glucose intolerance and adipose tissue inflammation in obese mice [21]. The underlying mechanisms likely include shifts towards maintenance and repair, enhanced stress resistance, and improved cellular health [22,23]. However, research on the impact of IF on reproductive health is relatively scarce. Existing studies have focused primarily on the effects of IF on reproductive hormone levels in men without obesity. These investigations exclusively employed daily 8 h TRE protocols, predominantly combined with triweekly resistance training sessions, in men who were young, healthy, and classified as non-obese Intriguingly, all studies consistently reported sustained reductions in both total testosterone concentrations and free androgen index (FAI) following 4–44 weeks of intervention, irrespective of TRE implementation as monotherapy or combined with exercise [24,25,26,27]. The impact of IF on the fertility of males with obesity remains largely unexplored.

Obesity-induced metabolic diseases are associated with disruptions across various interconnected ‘omic’ layers, including the transcriptome and metabolome. Omics-based analyses offer valuable insights into the systemic effects of obesity, including its impact on non-high metabolic rate organs (HMROs), such as male reproductive organs [28,29,30]. However, despite these advancements, studies that integrate multiple omics approaches to explore the dynamic molecular interactions involved in the alleviation of obesity-related adverse effects by IF are lacking.

Consequently, the primary objective of this study was to investigate the impact of IF on the metabolic profiles of obese mice, with an emphasis on its potential advantages for enhancing fertility outcomes. By combining transcriptomic and metabolomic analyses, the aim of this study was to provide a deeper understanding of the comprehensive benefits of IF as a strategy to mitigate the extensive effects of obesity.

## 2. Methods

### 2.1. Animals

**Figure 1:**
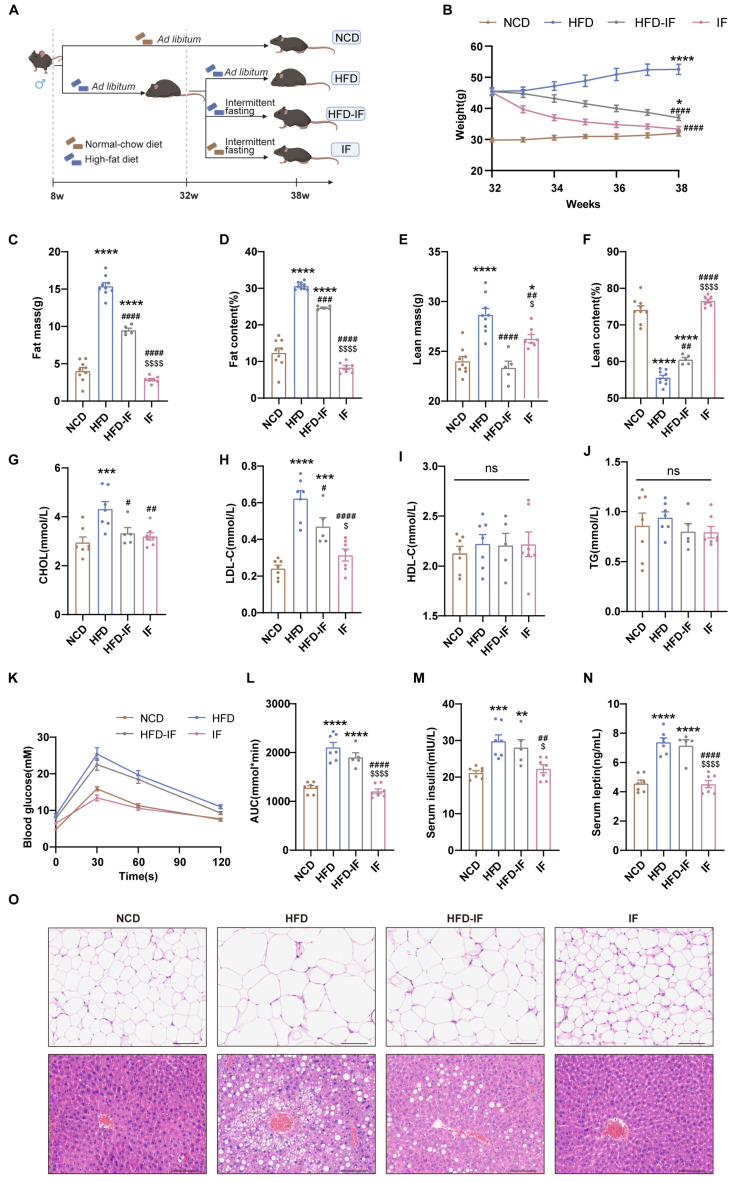
Mitigation of intermittent fasting on high-fat-diet induced obesity in mice. (**A**) Schematic diagram of the experimental procedures. (**B**) Body weight (n = 5–9 mice per group). (**C**–**F**) Body composition (n = 5–9 mice per group). (**G**–**J**) Serum lipid levels (n = 5–7 mice per group). (**K**) GTT and (**L**) GTT AUC values. (n = 5–7 mice per group). (**M**) Overnight fasting blood insulin and (**N**) leptin levels (n = 5–7 mice per group). (**O**) Gonadal WAT and liver H&E staining (scale bar, 100 μm) (n = 5 mice per group). Results shown as mean ± SEM. One-way ANOVA was used for comparing the differences among indicated groups (* *p* < 0.05, ** *p* < 0.01, *** *p* < 0.001, and **** *p* < 0.0001 vs. NCD; ^#^ *p* < 0.05, ^##^ *p* < 0.01, ^###^ *p* < 0.001, and ^####^ *p* < 0.0001 vs. HFD; ^$^ *p* < 0.05, ^$$$$^ *p* < 0.0001 vs. HFD-IF; ns, not significant).

Male C57BL/6J mice, aged 8 weeks, were acquired from Shanghai Model Organisms, Shanghai, China. The Animal Ethics Committee of Shanghai Model Organisms approved the experimental protocol (Protocol Number: IACUC 2023-0014-06). The mice were group-housed under a constant 12:12 h light/dark cycle and an ambient temperature of 23 °C. All experiments involved age-matched male mice. As designed in the previous study [11,31], the mice were fed ad libitum with either a normal chow diet (NCD) consisting of 10% fat, 70% carbohydrate, and 20% protein (Xietong 1010088, Jiangsu, China, *n* = 9) or a high-fat diet (HFD) comprising 60% fat, 20% carbohydrate, and 20% protein (Research Diets D12492, New Brunswick, NJ, USA, *n* = 21) for 24 weeks to induce obesity. At 32 weeks of age, a subset of mice in the HFD group (*n* = 12) were subjected to IF, forming two additional groups: the HFD-IF group (*n* = 5), which alternated between fasting and a HFD every other 24 h period, and the IF group (*n* = 7), which alternated between fasting and a NCD (Figure 1A). Both IF regimens began at 5:00 PM, 2 h before the night cycle. During the fasting period, the mice had free access to water. Body weights were recorded weekly at the beginning of each fasting day.

### 2.2. Body Composition Analysis

Body composition was assessed using a body composition analyzer (QMR06-090H, Hangzhou, China) at the completion of the intervention. All measurements were performed in the fed state, with individual values recorded for fat mass, fat content, lean mass, and lean content per mouse.

### 2.3. Reproductive Phenotype Assessment

At week 38, male mice were paired with 12-week-old females (three to four females per male) for 5 days. Females were checked daily for copulatory plugs as an indicator of mating. Plugged females were isolated in individual cages, and pregnancy was confirmed 12 days later. Litter sizes were recorded on the delivery day.

### 2.4. Glucose Tolerance Test (GTT)

At week 38, GTTs were conducted for the mice in all groups, with tests on mice in the HFD-IF and IF groups performed during fasting days without food replacement afterwards. The mice were fasted for 16 h with access to water. Baseline blood samples were taken from fully conscious mice via tail snip, followed by an intraperitoneal glucose injection (0.75 g/kg). Blood glucose levels were measured via tail vein blood at 0, 30, 60 and 120 min postinjection using the glucometer (Roche, Basel, Switzerland).

### 2.5. Serum and Tissue Collection

At the completion of the intervention, following an overnight fast, the mice were deeply anesthetized with isoflurane (Ruiwode R510-22, Shenzhen, China) and blood was collected from the retro-orbital venous plexus. Subsequently, euthanasia was performed by cervical dislocation under sustained anesthesia to ensure death prior to tissue collection. The blood was allowed to clot for 2 h at room temperature and was then centrifuged at 2000 rpm for 15 min at 4 °C to obtain the serum. Following euthanasia, liver, gonadal white adipose tissue (WAT), testes and cauda epididymides were collected. The testes were weighed individually. The left testis and gonadal WAT were prepared for histological examination, while the right testis and gonadal WAT were stored at −80 °C. The cauda epididymides are used to collect sperm.

### 2.6. Serum Hormone and Lipid Analysis

We measured insulin, leptin, estradiol, and testosterone levels in the serum using enzyme linked immunosorbent assay (ELISA) kits (Chengzhi Kewei, Beijing, China) according to the manufacturer’s instructions. Additionally, we measured serum lipid levels including total cholesterol (CHOL), LDL-C, high-density lipoprotein cholesterol (HDL-C), and triglycerides (TG) using an automatic biochemical analyzer (Toshiba 120, Tokyo, Japan).

### 2.7. Histological Examination

Livers, testes and gonadal WAT were fixed in 4% paraformaldehyde for 24 h, dehydrated in ethanol, embedded in paraffin, and sectioned (5 µm thickness). The sections were stained with haematoxylin and eosin (H&E) and observed under a microscope (Olympus Ex61, Tokyo, Japan).

### 2.8. Sperm Analysis

The left cauda epididymis from each animal was placed in a dish containing 1 mL of human tubal fluid (HTF) media (Millipore MR-070-D, Burlington, MA, USA) and incubated at 37 °C for 45–60 min to allow sperm to flow out to obtain a sperm suspension. Sperm counts were performed using a haemocytometer (Hawksley, Lancing, UK) under a light microscope [32] (Nikon ECLIPSE E100, Tokyo, Japan). To assess motility, 10 µL of sperm suspension was examined under a microscope at 400× magnification. At least 200 sperm were counted, and motility was recorded as the percentage of progressively motile sperm. The morphology of the sperm was evaluated by staining using a Diff-Quick kit (Jian-Cheng, Nanjing, China). Three microscopic fields from each sample were randomly chosen, and defective spermatozoa were reported as a percentage of the total count [33].

### 2.9. In Vitro Fertilization and Early Embryo Development

IVF was performed using established methods [34]. The right cauda epididymis from each animal was placed in a dish containing 200 µL of capacitation-toyoda yokoyama hoshi (c-TYH) medium (Sudgen 72021, Nanjing, China) and incubated at 37 °C for 30 min to obtain capacitated sperm. C57BL/6J female mice, aged 3.5–4 weeks, received intraperitoneal injections of 5 IU pregnant mare serum gonadotropin (PMSG) (San-Sheng, Shanghai, China) 48 h apart, followed by 5 IU human chorionic gonadotropin (hCG) (San-Sheng, China) to induce superovulation. Cumulus-oocyte complexes (COCs) were collected from ampullae 13–14 h post hCG injection and incubated overnight in HTF medium with capacitated sperm. The embryos were then washed and cultured in potassium simplex optimization medium (KSOM Millipore, Burlington, MA, USA) supplemented with mineral oil (Dong-yun, Guangzhou, China) at 37 °C in 5% CO_2_ and 100% humidity until they reached the blastocyst stage. Embryo developmental competence was quantified using two key metrics: (1) 2-cell formation rate, calculated as (2-cell embryos/total zygotes cultured) × 100% at 24 h postincubation; and (2) blastocyst formation rate, determined by (blastocysts/total zygotes) × 100% at 96 h postincubation.

### 2.10. RNA Sequencing

Total RNA was extracted from testis tissues using a TRIzol reagent kit (Takara, Shiga, Japan). The mRNA was then enriched using oligo (dT) beads and fragmented in fragmentation buffer before being reverse transcribed into cDNA using random hexamers (Takara 3801, Shiga, Japan) to ensure unbiased coverage of RNA fragments. Following RNA library construction, the RNA concentration was accurately measured using a Qubit instrument (Invitrogen, Carlsbad, CA, USA) to ensure a minimum concentration of 500 ng/μL. Paired-end sequencing was performed on the Illumina NovaSeq 6000 platform (Illumina Inc., San Diego, CA, USA), generating reads of 151 bp. Differential gene expression analysis was conducted using DESeq2, which is based on a negative binomial distribution. Genes with a |log2(fold change)| > 1 and *p* value < 0.05 were filtered as differentially expressed genes (DEGs). Gene Ontology (GO) and Kyoto Encyclopedia of Genes and Genomes (KEGG) analyses were carried out with the ‘ClusterProfiler’ R package (version 3.18.1).

### 2.11. Untargeted Metabolomic Analysis by LC-MS

Serum and testicular metabolites were analyzed as described in a recent study [35]. Samples were thawed on ice; 50 μL of serum and 25 mg of testis tissue from the NCD, HFD, and IF groups were prepared for LC-MS analysis. Briefly, sample extraction was performed using extraction buffer, which was a mixture of methanol, acetonitrile and ddH_2_O (2:2:1, *v*/*v*). A pooled quality control sample was prepared by mixing equal volumes of each extracted supernatant. The samples (2 μL) were injected into a Vanquish UHPLC system (Thermo, Waltham, MA, USA) equipped with an ACQUITY UPLC BEH Amide column (2.1 mm × 50 mm, 1.7 μm; Waters, Milford, MA, USA). The sample plate temperature was 4 °C. Mobile phase A consisted of a water mixture with 25 mmol/L ammonium acetate and 25 mmol/L ammonium hydroxide, while mobile phase B consisted of acetonitrile. The electrospray ionization conditions of the Orbitrap Exploris 120 mass spectrometer (Xcalibur, version: 4.4, Thermo, Waltham, MA, USA) were set as follows: sheath gas flow rate, 50 Arb; aux gas flow rate, 15 Arb; capillary temperature, 320 °C; full MS resolution, 60,000; MS/MS resolution, 15,000; collision energy, SNCE 20/30/40; and spray voltage, 3.8 kV (positive) or −3.4 kV (negative).

Raw data were preprocessed, and multivariate analysis for metabolite profiling was performed using ProteoWizard (version 3.0.21229) and R (version 4.4.1). Partial least squares discriminant analysis (PLS-DA) and orthogonal partial least squares discriminant analysis (OPLS-DA) were used to profile global metabolic changes. Variable importance in projection (VIP > 1.0) combined with an unpaired Student’s *t* test (*p* < 0.05) was used to identify the differentially abundant metabolites. MetaboAnalyst 5.0 was used for the analysis of metabolic pathways.

### 2.12. Real-Time Quantitative PCR

Total RNA was extracted from testes and liver tissues using TRIzol reagent (Takara, Shiga, Japan) and reverse transcribed into cDNA using a PrimeScript RT Reagent Kit (Takara RR037A, Shiga, Japan). Quantitative real-time PCR was conducted on a 7900HT Fast Real-Time PCR System (Applied Biosystems, Foster City, CA, USA) with SYBR Premix Ex Taq (Takara RR420L, Shiga, Japan). mRNA levels were quantified using the 2^−ΔΔCt^ method, with β-actin serving as an internal control. The primers used for qPCR were as follows: Uox forward, 5′-GAAGTGGAATTTGTCCGAACTGG-3′; Uox reverse, 5′-CGAAGTTGCCACCTCTTTGAT-3′; β-actin forward, 5′-GGCTGTATTCCCCTCCATCG-3′; and β-actin reverse, 5′-CCAGTTGGTAACAATGCCATGT-3′.

### 2.13. Western Blot Analysis

After thawing on ice, mouse liver tissues were homogenized in RIPA buffer with a protease inhibitor cocktail. Proteins were separated using 10% SDS-PAGE and transferred to PVDF membranes. The membranes were blocked with 5% nonfat milk and incubated with primary antibodies against Uox (1:500; Santa Cruz sc166214, Dallas, TX, USA) and α-tubulin (1:2000; Proteintech 66031-1-1g, Wuhan, China) overnight at 4 °C. The membranes were then incubated with a horseradish peroxidase-conjugated secondary anti-mouse antibody (1:5000, Abcam ab6728, Cambridge, UK). Immunoblots were visualized with an iBright FL1000 (version 5.4.0) and analyzed via ImageJ software (version v1.54p).

### 2.14. Immunohistochemistry (IHC) Analysis

Paraffin-embedded liver sections (4 µm) were deparaffinized, rehydrated, and subjected to antigen retrieval in citrate buffer (pH 6.0) via microwave heating. Endogenous peroxidase was blocked with 3% H_2_O_2_ (25 min), followed by 3% BSA blocking (30 min). Sections were incubated with rabbit anti-Uox antibody (1:50; Santa Cruz, sc166214) at 4 °C overnight, then with HRP-conjugated secondary antibody (DAKO K5007, Glostrup, Denmark ready-to-use) for 50 min at room temperature. DAB visualization, hematoxylin counterstaining, dehydration, and neutral balsam mounting were performed. Uox-positive areas were quantified using ImageJ.

### 2.15. Statistical Analysis

The data are presented as means ± SEMs. One-way ANOVA was used to compare the differences among indicated groups. The Gehan–Breslow–Wilcoxon test was applied to compare the percentage of plugged females between groups. Data visualization was performed using GraphPad Prism 9.0.0. *p* < 0.05 was considered to indicate statistical significance, with specific notations (* *p* < 0.05, ** *p* < 0.01, *** *p* < 0.001, and **** *p* < 0.0001 vs. NCD; ^#^ *p* < 0.05, ^##^ *p* < 0.01, ^###^ *p* < 0.001, and ^####^ *p* < 0.0001 vs. HFD) indicating levels of significance. Statistical methods and *p* values are detailed in the figure legends.

## 3. Results

### 3.1. IF Mitigates HFD-Induced Obesity in Mice

Compared with those of mice in the NCD group, the body weights of mice in the HFD group were greater; however, intermittent fasting reduced this increase. Mice in the HFD-IF group remained heavier than mice in the NCD group, while the weight of the mice in the IF group was comparable to that of mice in the NCD group (Figure 1B). Compared with mice in the NCD group, mice in the HFD group presented increased fat mass and reduced lean content. The mice in the HFD-IF group showed partial normalization of body composition parameters, whereas the body composition parameters for mice in the IF group closely matched those of mice in the NCD group (Figure 1C–F). Additionally, HFD-induced hyperlipidemia persisted in mice in the HFD-IF group, especially elevated LDL-C, while in mice in the IF group, levels normalized to those in the control group (Figure 1G–J). The glucose area under the curve (AUC) and serum insulin and leptin levels in the HFD and HFD-IF groups were greater than those in the NCD group, while these parameters in the IF group were not significantly different from those in the NCD group (Figure 1K–N). Compared with HFD-IF, IF better prevented fat accumulation in gonadal adipocytes and hepatic steatosis (Figure 1O). These results suggest that IF can counteract obesity but that its efficacy is reduced when it is combined with a continued HFD, whereas a return to a normal diet yields stronger antiobesity effects.

### 3.2. IF Alleviates HFD-Induced Male Infertility in Mice

**Figure 2:**
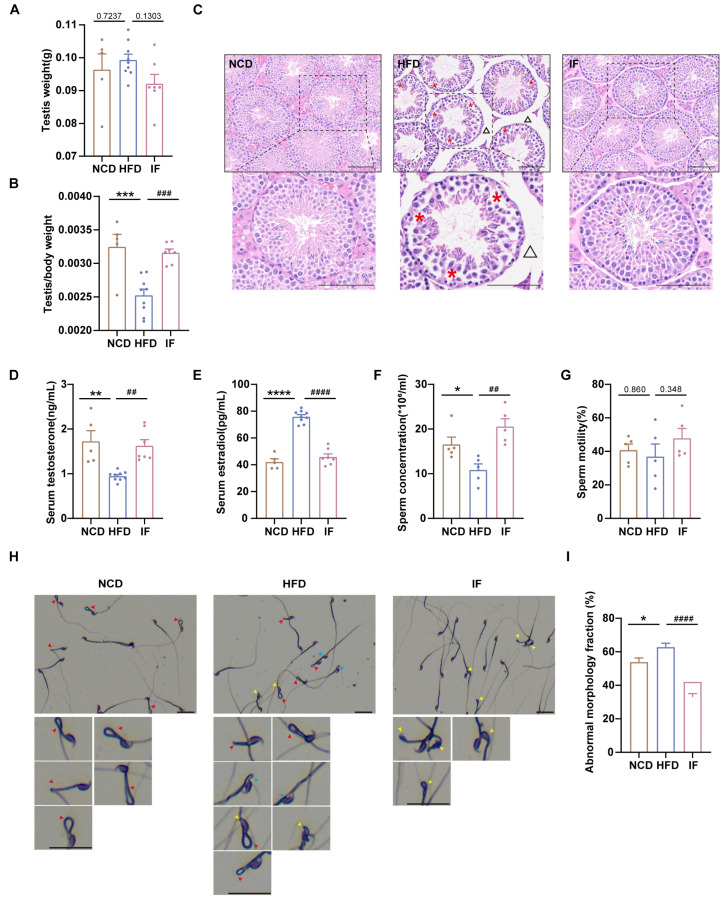
Amelioration of intermittent fasting on high-fat-diet induced abnormal testicular morphology, reproductive hormones and sperm parameter. (**A**) Testis weight (n = 5–9 mice per group). (**B**) Testis index = testis weight/body weight (n = 5–9 mice per group). (**C**) H&E staining was used to show testicular morphology (n = 5–9 mice per group; scale bar, 100 μm). The red asterisk revealed loosely arranged spermatogenic cells on the seminiferous epithelium in HFD mice and the black triangle indicates enlarged spacing between adjacent seminiferous tubules. (**D**) Serum testosterone levels (n = 5–9 mice per group). (**E**) Serum estradiol levels (n = 5–9 mice per group). (**F**) Sperm concentration and (**G**) motility (n = 5 mice per group). (**H**) Representative images of sperm morphology (scale bar, 30 μm). The red, yellow and blue arrows represent sperm midpiece defects, head defects and neck defects, respectively. (**I**) The fraction of sperm abnormal morphology. (* *p* < 0.05, ** *p* < 0.01, *** *p* < 0.001, and **** *p* < 0.0001 vs. NCD; ^##^ *p* < 0.01, ^###^ *p* < 0.001, and ^####^ *p* < 0.0001 vs. HFD).

To assess the impact of IF on reproductive organ morphology, we first weighed the testes and normalized them to body weight. The weights of the testes were not significantly different among the three groups, whereas the relative testis weights were greater for mice in the IF group than for mice in the HFD group (Figure 2A,B). H&E analysis revealed that testes in the HFD group had loosely arranged spermatogenic cells within the tubules and expanded interstitial spaces between adjacent tubules; in contrast, testes in the IF group exhibited restored testicular structure to nearly normal levels (Figure 2C). We also analyzed testosterone and estradiol levels in blood serum. Compared with mice in the NCD group, mice in the HFD group presented significantly higher testosterone and elevated estradiol levels; however, in the IF group, mice exhibited levels of reproductive hormones similar to those observed in control mice (Figure 2D,E). While the sperm concentration was lower in the HFD group, in the IF group, it was notably increased, although sperm motility did not significantly differ across the groups (Figure 2F,G). Sperm morphology, assessed using Diff-Quick staining, revealed a greater rate of total abnormalities (including sperm head defects, neck defects and midpiece defects) in mice in the HFD group than in mice in the control group; in mice in the IF group, there was a significantly lower rate of abnormalities (Figure 2H,I). These results indicate that IF mitigates the adverse effects of a HFD on testicular morphology, reproductive hormones, and sperm parameters.

**Figure 3:**
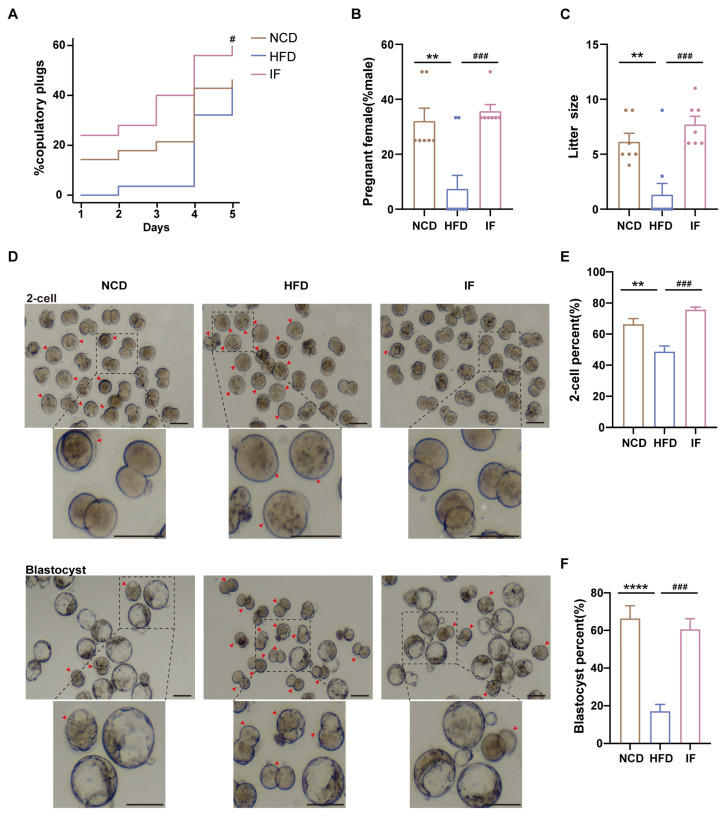
Effects of intermittent fasting on reproductive success and early embryo development in high-fat-diet mice. (**A**) The percentage of plugged and (**B**) pregnant females after mating with males (n = 7–9 mice per group; 3–4 females/male). (**C**) The size of litters (n = 7–9 mice per group). (**D**) Representative images of 2-cells and blastocysts development (Scale bar 100 um). Arrows showed early embryos that had not developed into 2 cells at 24 h and blastocysts at 96 h after in vitro fertilization, respectively. (**E**,**F**) The rate of 2-cells and blastocysts development. (n = 5 mice per group). Results shown as mean ± SEM. Gehan-Breslow-Wilcoxon test was used for comparing the different percentage of plugged females between HFD vs. NCD and IF vs. HFD. One-way ANOVA was used for comparing the differences among indicated groups (** *p* < 0.01 and **** *p* < 0.0001 vs. NCD; ^#^ *p* < 0.05 and ^###^ *p* < 0.001 vs. HFD).

Mating trials revealed enhanced copulatory success, greater pregnancy rates, and larger litter sizes in the IF group than in the HFD group (Figure 3A–C). Further analysis of IF’s effects on early embryo development through in vitro fertilization demonstrated that IF effectively ameliorated the impaired developmental rates caused by HFD exposure. This restoration was evidenced by significant improvements in both 2-cell embryo formation rate (24 h incubation) and blastocyst formation rate (96 h incubation). (Figure 3D–F). These results indicate that IF enhances reproductive success and early embryo development in mice adversely affected by a HFD.

### 3.3. IF Reverses Testis Metabolomic Changes in HFD-Fed Mice

**Figure 4:**
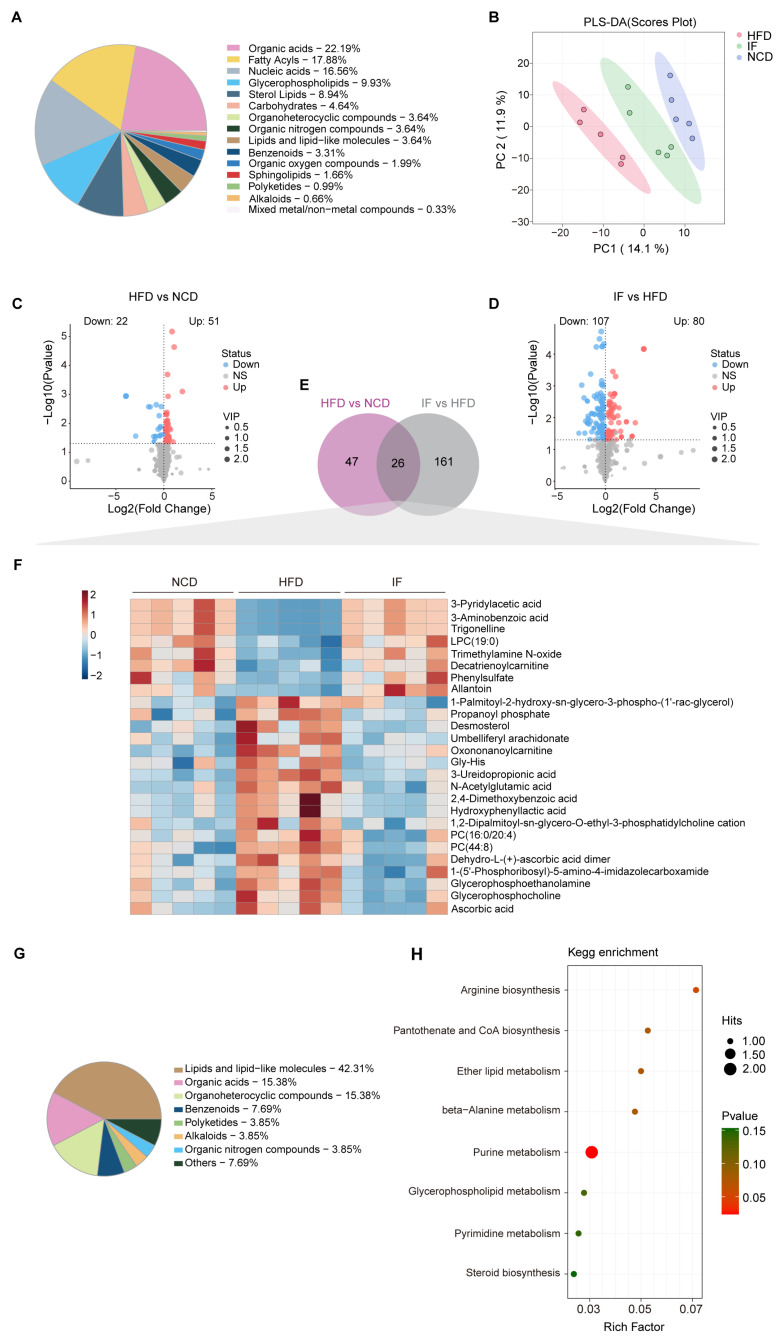
Intermittent fasting reverses high-fat-diet induced changes in testis metabolites. (**A**) Metabolites composition of testis samples from indicated groups. (**B**) Scatter plot of PLS-DA for metabolites of mice in the indicated groups. (**C**) Volcano plot of significant differential metabolites of HFD vs. NCD and (**D**) IF vs. HFD. (**E**) Venn plot showing the overlap of differential metabolites shown in (**C**,**D**). (**F**) Relative content heatmap for overlapped metabolites of mice in the indicated groups. (Scale bar shows Z-score; n = 5 mice per group). (**G**) Composition of overlapped metabolites in the Venn plot. (**H**) KEGG bubble plot of metabolites enrichment reversed by intermittent fasting. The horizontal coordinate is the extent to which the pathway is affected. Size of dots represents number of metabolites in each KEGG pathway. The *p*-values calculated by the enrichment analysis are described in terms of their color intensity.

LC-MS analysis was carried out using testicular tissue to examine changes in metabolites within the reproductive system. A total of 699 metabolites were annotated, with organic acids (22.2%), fatty acyls (17.9%), and nucleic acids (16.6%) being the most abundant categories (Figure 4A). PLS-DA revealed that the metabolic profile of the IF group was more similar to that of the NCD group than to that of the HFD group (Figure 4B). OPLS-DA further confirmed the distinct metabolic differences between the HFD and NCD groups and between the IF and HFD groups. The reliability of these models was supported by R^2^Y and Q^2^ values of 0.889 and 0.965 for HFD vs. NCD and 0.502 and 0.776 for IF vs. HFD, respectively (Supplementary Figure S1A,B). Using VIP > 1 and *p* value < 0.05, 73 and 187 differentially abundant metabolites were identified in the comparisons between the NCD and HFD groups and between the IF and HFD groups, respectively (Figure 4C,D). KEGG enrichment analysis revealed that the differentially abundant metabolites between the HFD and NCD groups were enriched mainly in nucleotide metabolism, arginine biosynthesis and lipid metabolism. The regulated metabolites in the comparison of the IF and HFD groups were involved mainly in purine metabolism, amino acid metabolism and carbohydrate metabolism. Interestingly, carbohydrate metabolism, such as fructose and mannose metabolism, pentose and glucuronate interconversions and galactose metabolism, was enriched only in the IF group compared with the HFD group (Supplementary Figure S1C).

Further analysis revealed that 26 differentially abundant metabolites between the HFD and NCD groups and between the IF and HFD groups overlapped (Figure 4E, Supplementary Table S1). A heatmap revealed that these 26 overlapping metabolites were induced by a HFD and reversed by IF (Figure 4F) and were associated mainly with classes of lipids and lipid-like molecules (42.3%), organic acids (15.4%), and organoheterocyclic compounds (15.4%) (Figure 4G). KEGG pathway enrichment analysis revealed that these 26 altered metabolites were involved in 8 metabolic pathways, with a significant emphasis on purine metabolism (Figure 4H).

### 3.4. IF Reverses Serum Metabolomic Changes in HFD-Fed Mice

**Figure 5:**
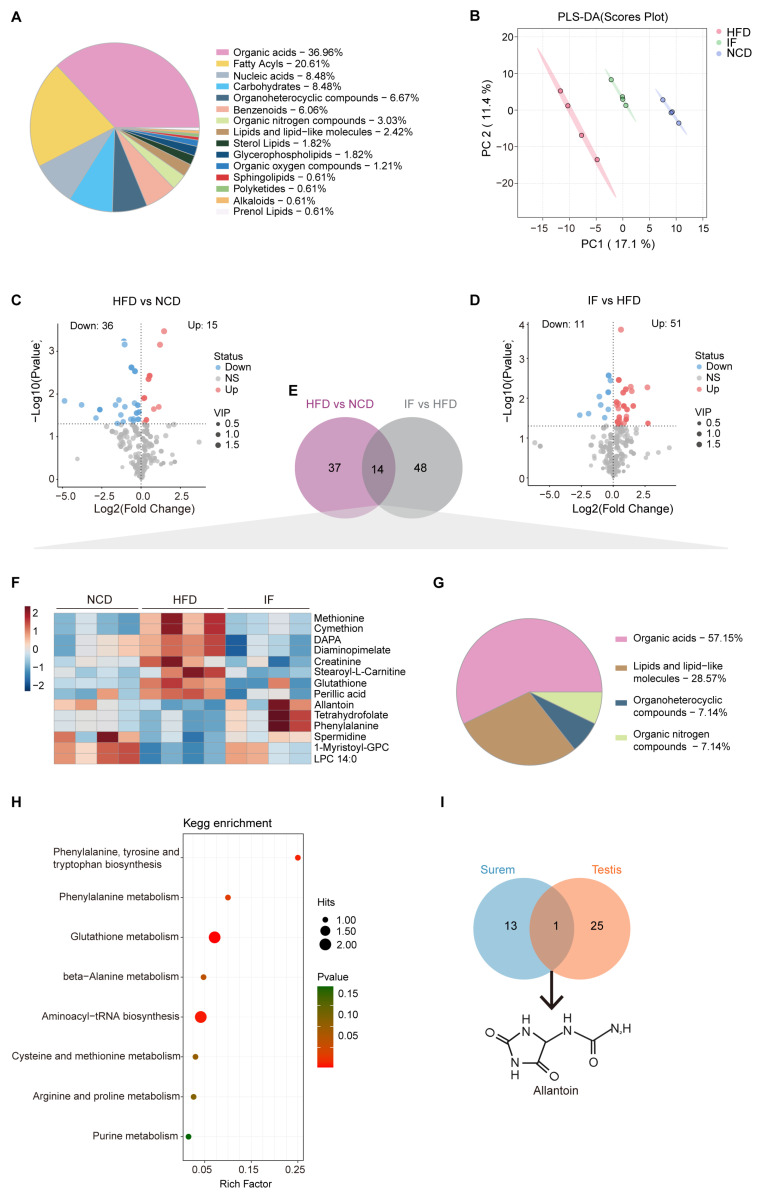
Intermittent fasting reverses high-fat-diet induced changes in serum metabolites. (**A**) Metabolites composition of serum samples from indicated groups. (**B**) Scatter plot of PLS-DA for metabolites of mice in the indicated groups. (**C**) Volcano plot of significant differential metabolites of HFD vs. NCD and (**D**) IF vs. HFD. (**E**) Venn plot showing the overlap of differential metabolites shown in (**C**,**D**). (**F**) Relative content heatmap for overlapped metabolites of mice in the indicated groups. (Scale bar shows Z-score; n = 4 mice per group). (**G**) Composition of overlapped metabolites in the Venn plot. (**H**) KEGG bubble plot of metabolites enrichment reversed by intermittent fasting. The horizontal coordinate is the extent to which the pathway is affected. Size of dots represents number of metabolites in each KEGG pathway. The *p*-values calculated by the enrichment analysis are described in terms of their color intensity. (**I**) Venn plot showed the overlap of intermittent fasting reversed metabolites in serum and testis samples. Arrow showed the structure of allantoin.

To evaluate circulating metabolic variations among the indicated groups globally, liquid chromatography–mass spectrometry (LC-MS) was performed using blood serum samples. A total of 363 metabolites were annotated, predominantly organic acids (37%), fatty acyls (20.6%), and nucleic acids (8.5%) (Figure 5A). Notably, PLS-DA highlighted distinct separations among the three groups. The serum metabolite profile of the IF group closely resembled that of the NCD group rather than the HFD group. These findings suggest that IF may partially reverse the alterations in serum metabolites induced by a HFD (Figure 5B). Subsequent analysis using OPLS-DA confirmed significant metabolic differences between the HFD and NCD groups, as well as between the IF and HFD groups. The interpretation ability parameters (R^2^Y) approached 1, and the prediction ability parameters of the model (Q^2^) were greater than 0.5, which indicated that the model had superior reliability and prediction ability. The R^2^Y and Q^2^ values in the HFD vs. NCD and IF vs. HFD comparisons were 0.965 and 0.956 and 0.717 and 0.694, respectively, indicating the reliability of the proposed models (Supplementary Figure S2A,B). Volcano plots displayed variable contributions (variable importance in the projection, VIP), fold changes (FCs), and *p* values for each metabolite, identifying 51 and 62 differentially abundant metabolites between the HFD and NCD groups and between the IF and HFD groups, respectively (VIP > 1, *p* < 0.05) (Figure 5C,D). The KEGG enrichment analysis revealed that the differentially abundant metabolites between the HFD and NCD groups and between the IF and HFD groups were enriched mainly in amino acid metabolism. Notably, only in the comparison of the IF and HFD groups was there specific enrichment in branched-chain amino acids (BCAA) metabolism, glycerolipid metabolism, and the synthesis and degradation of ketone bodies (Supplementary Figure S2C).

Among the differentially abundant metabolites, 14 overlapped (Figure 5E, Supplementary Table S2). A heatmap revealed that the 14 overlapping metabolites were induced by a HFD and reversed by IF (Figure 5F). Among them, organic acids, lipids, lipid-like molecules, organoheterocyclic compounds, and organic nitrogen compounds accounted for 57.1%, 28.6%, 7.1% and 7.1%, respectively (Figure 5G). KEGG pathway enrichment analysis revealed that the 14 altered metabolites were involved in 8 metabolic pathways, with 6 related to amino acid metabolism and 2 linked to translation and purine metabolism (Figure 5H).

A comparison of the reversed differentially abundant metabolites in the serum (14 metabolites) and testis (26 metabolites) samples revealed a consistent trend: one metabolite, allantoin—the end product of purine catabolism, oxidized from uric acid by the action of urate oxidase (Uox)—was decreased in the HFD group and increased in the IF group (Figure 5I). These findings indicate that IF significantly affects both serum and testis metabolism in obese mice, predominantly by modulating purine metabolism.

### 3.5. IF Reverses the Testis Transcriptome in HFD-Fed Mice

**Figure 6:**
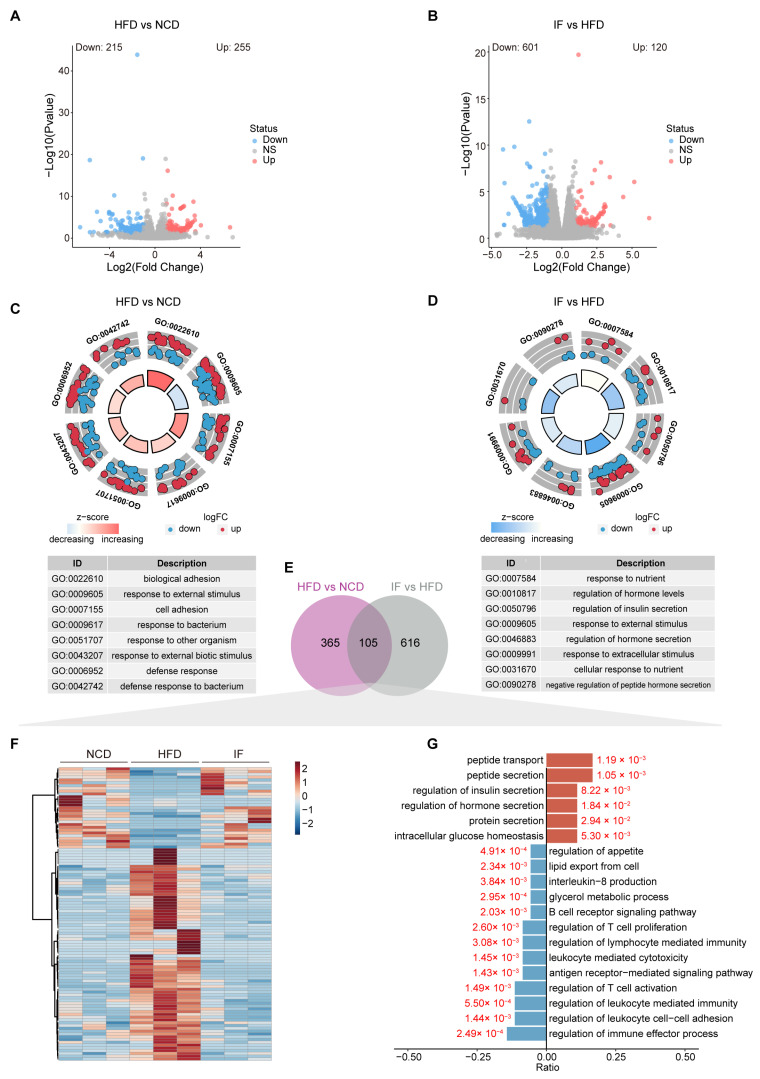
Intermittent fasting reverses the testis transcriptome in high-fat-diet mice. (**A**) Volcano plot of significant DEGs of HFD vs. NCD and (**B**) IF vs. HFD. (**C**) Circle diagram of GO enrichment analysis of HFD vs. NCD and (**D**) IF vs. HFD. (**E**) Venn plot showing the overlap DEGs shown in (**A**,**B**). (**F**) Relative content heatmap for overlapped DEGs of mice in the indicated groups (Scale bar shows Z-score; n = 3 mice per group). (**G**) GO enrichment analysis of overlapped downregulated (blue bars: Log2FC < 1, *p* value < 0.05) and upregulated (red bars: Log2FC > 1, *p* value < 0.05) genes.

To explore the relationship between metabolic benefits and gene expression changes, we conducted RNA sequencing analysis using testicular tissue. The analysis revealed 570 DEGs between the HFD group and the NCD group, with 215 genes downregulated and 255 genes upregulated (Figure 6A). A total of 721 DEGs were identified between the IF group and HFD group, with 601 downregulated and 120 upregulated genes (Figure 6B). GO enrichment analysis highlighted significant functional changes due to dietary factors and IF intervention. Biological adhesion (GO:0022610), response to external stimulus (GO:0009605), and cell adhesion (GO:0007155) were the main biological processes enriched in the HFD vs. NCD group comparison (Figure 6C). The terms response to nutrients (GO:0007584), regulation of hormone levels (GO:0010817), and regulation of insulin secretion (GO:0050796) were enriched in the IF vs. HFD group comparison (Figure 6D).

In our analyses comparing the HFD and NCD groups and the IF and HFD group, we identified 105 DEGs present in both comparisons (Figure 6E, Supplementary Table S3). A heatmap analysis revealed that the DEGs induced by a HFD were reversed following IF treatment, with 26 genes upregulated and 79 downregulated (Figure 6F). GO analysis conducted separately for the upregulated and downregulated DEGs revealed distinct pathways affected by IF. The upregulated genes were involved primarily in peptide metabolism, the regulation of insulin and hormone secretion, and the maintenance of intracellular glucose levels. In contrast, the downregulated genes were associated with lipid metabolism, inflammation, and immune response pathways; these pathways included lipid export, glycerol metabolism, interleukin-8 production, B cell receptor signaling, T cell proliferation regulation, and lymphocyte-mediated immunity (Figure 6G). These findings suggest that IF can counteract the detrimental effects of a HFD on reproductive function by normalizing glucose and lipid balance in the testes and mitigating immune system disturbances.

### 3.6. Integrated Analysis Reveals That IF Is Associated with Allantoin Production

**Figure 7:**
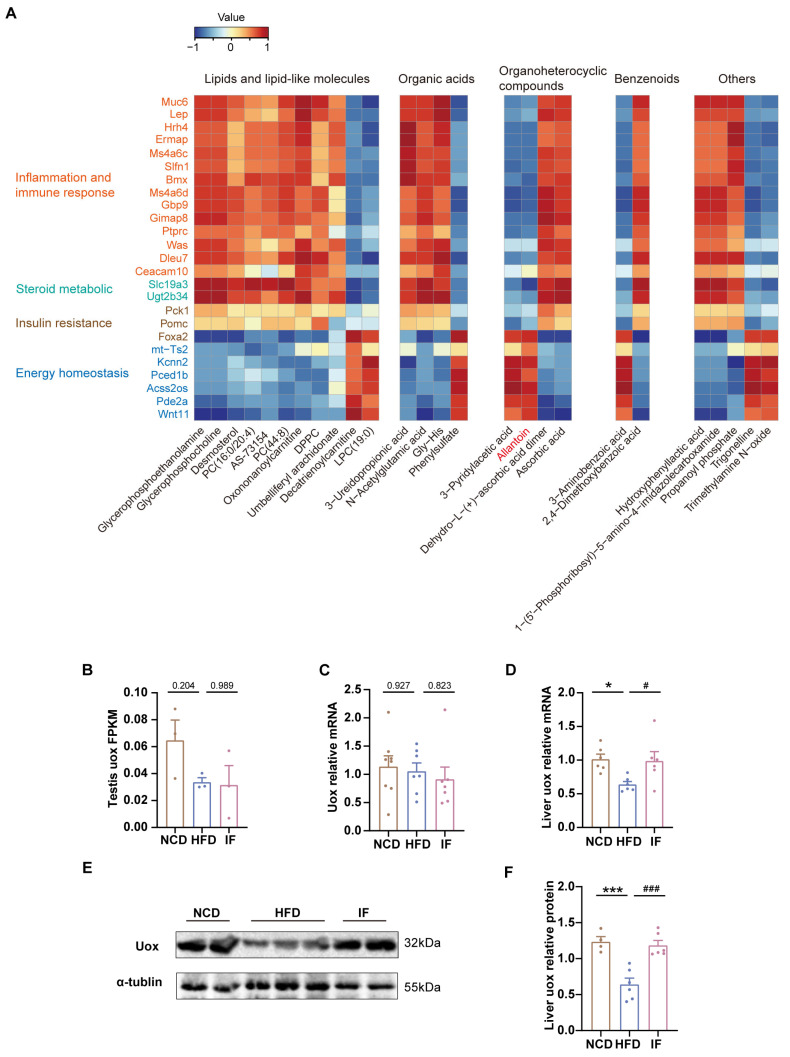
Integrated analysis of metabolites and genes reversed by intermittent fasting in testis. (**A**) Heatmap of Spearman’s correlation coefficients between changes in reversed genes and metabolite alterations caused by intermittent fasting (Scale bar shows Z-score). (**B**) RNA-seq results of uox expression levels in testis (n = 3 mice per group). (**C**) RT-qPCR results of uox expression levels in testis (n = 7–8 mice per group). (**D**) RT-qPCR results of uox expression levels in liver (n = 6 mice per group). (**E**) WB analysis of uox expression levels in liver and (**F**) quantified by ImageJ software (n = 4–6 mice per group). Results shown as mean ± SEM. The one-way ANOVA test was used for comparing the differences among indicated groups (* *p* < 0.05 and *** *p* < 0.001 vs. NCD; ^#^ *p* < 0.05 and ^###^ *p* < 0.001 vs. HFD).

To delineate the interplay between differentially expressed genes and abundant metabolites reversed by IF, Pearson correlation analysis was employed. We found that most lipids and organic acids were positively associated with inflammation, immune responses, steroid metabolism, and insulin resistance but negatively associated with energy homeostasis (Figure 7A). Notably, elevated allantoin levels were inversely correlated with genes linked to inflammation and immune responses (e.g., Muc6, Lep, and Gbp9) but positively correlated with genes critical to glycolipid and energy balance (e.g., Foxa2, mt-Ts2, and Wnt11). These findings indicate that IF might bolster lipid metabolism and insulin sensitivity, diminish inflammation and immune reactions, and stabilize energy levels by increasing allantoin concentrations in the testis, potentially leading to increased fertility.

To explore how IF affects the level of allantoin within the testis, we initially investigated the expression of Uox, an enzyme crucial for allantoin production, in the testis. Notably, neither testicular RNA-seq nor qPCR revealed that IF significantly increased the expression of Uox (Figure 7B,C). This finding suggests that the increase in testicular allantoin might not stem from enhanced urate oxidase expression within the testis itself. Given that IF also increases serum allantoin levels and considering the primary role of the liver in purine metabolism, it is plausible to hypothesize that IF increases hepatic Uox expression; this increase would then lead to elevated levels of hepatic allantoin, which could travel via the bloodstream to the testis and exert beneficial effects there.

To test that hypothesis, we performed RT-PCR analysis of liver Uox mRNA expression and confirmed that the HFD-induced downregulation of Uox expression was reversed, as shown by IF (Figure 7F). Consistently, IF notably increased the protein levels of Uox in the liver (Figure 7E,F, Supplementary Figure S3). These results further suggest that IF promotes allantoin production through increased Uox expression in the liver, subsequently improving testicular metabolic homeostasis via the circulatory system.

## 4. Discussion

Our study demonstrated that IF mitigated obesity and its metabolic complications but that these benefits were contingent on dietary composition during the feeding period. While IF reduced body weight, normalized body composition, and lowered total cholesterol across groups, IF combined with a high-fat diet (HFD-IF group) markedly blunted those effects, failing to normalize LDL-C, fasting insulin, and leptin levels or improve glucose tolerance. Those findings parallel concerns raised in IF clinical trials, in which the unrestricted consumption of energy-dense foods during non-fasting periods undermines therapeutic outcomes [36]. Importantly, this metabolic inefficacy explains our decision to exclude a HFD-IF group for fertility analyses. Obesity-associated metabolic dysfunction is closely linked to male infertility (e.g., via oxidative stress, hormonal imbalances and systemic inflammation [37,38]. For example, obesity-related hyperleptinemia disrupts the HPG axis, leading to elevated LH and FSH levels, decreased testosterone levels, and abnormal sperm morphology [39]. Elevated leptin levels also reduce reproduction in obesity through the effect of leptin on the kisspeptin-GnRH pathway while simultaneously compromising Sertoli cell function and Leydig cell testosterone synthesis, ultimately promoting oxidative stress and sperm dysfunction [40]. Notably, mice in the HFD-IF group maintained elevated serum leptin levels postintervention, limiting the potential of IF to alleviate obesity-related infertility. For more effective weight management and fertility enhancement, we recommend fasting protocols combined with a reduction in high-calorie food intake.

The testicular microenvironment is important for spermatogenesis [41]; therefore, we performed testis metabolomics to explore the effects of IF on male fertility. IF reversed the 26 metabolite alterations induced by a HFD, primarily in terms of lipids and organic acids, with the levels of most metabolites significantly reduced. Notably, lipidomic remodeling may extend to steroidogenesis—testosterone (T) and dihydrotestosterone (DHT) synthesis critically depends on cholesterol availability and lipid transporters [42], suggesting IF’s metabolic regulation could concurrently support androgen production. Testicular lipid metabolism, especially phospholipid metabolism, is vital for reproductive health, as the testes have a high demand for lipid membranes due to the proliferation of germ cells [43]. In our study, we observed elevated levels of glycerophosphocholine (GPC) and glycerophosphoethanolamine (GPE) in HFD-fed mice, indicating an increase in phospholipid degradation, which may negatively affect spermatogenesis [44]. IF was found to reduce GPC and GPE levels, likely by preserving membrane integrity, which supports spermatogenesis and enhances sperm motility and fertilization efficiency. This membrane stabilization could also benefit epididymal function, as appropriate phospholipid composition is essential for maintaining epididymal fluid viscosity and sperm maturation microenvironment [45]. Moreover, the antioxidative capacity of IF was supported by the observed decrease in oxidative damage and in organic acids. Notably, ascorbic acid levels were elevated in the HFD group, indicating increased oxidative stress [46], which was significantly reduced by IF. These findings highlight the potential of IF in alleviating HFD-induced oxidative damage and inflammation. In addition to those pathways, IF uniquely enhanced the catabolic metabolism of carbohydrates in the testes, as evidenced by increased levels of metabolic products such as glucose-6-phosphate, fructose-6-phosphate, glucose-1-phosphate, and galactose-1-phosphate. Given that glucose metabolism is a primary energy source for spermatogenesis in the testes, reductions in glycolysis have been shown to inhibit spermatogenesis in rats [47]. Furthermore, recent studies have indicated that obesity can lead to the reduced expression of glucose transport proteins (GLUTs), impairing the glycolytic pathway in the testes and leading to male infertility [48]. Our present study revealed that IF may increase carbohydrate metabolism to correct the metabolic imbalance caused by a HFD, providing energy for spermatogenesis; this mechanism underlies the significant improvements in sperm parameters and morphology observed after IF. Overall, these testicular metabolic changes provide new insights into the role of IF in correcting obesity-related metabolic imbalances in the reproductive system.

Our serum metabolome analysis confirmed that a HFD led to dysregulated glycolipid metabolism and an increase in inflammatory metabolites and that IF, however, was able to reverse those alterations, indicating a comprehensive outlook for understanding how IF alters sperm parameters and increases male fertility. Specifically, IF enhances BCAA production, promoting glucose metabolism, fatty acid oxidation, and ketone body production, which can support systemic metabolism, thereby improving sperm parameters [49,50]. Additionally, IF reduces methionine levels while increasing spermidine levels, which are linked to improved lipid profiles and glucose tolerance [51,52]; it also normalizes elevated glutathione levels, mitigating oxidative stress and inflammation [53]. Testicular and serum metabolites were different but well correlated. Our results demonstrated that IF primarily decreased organic acid levels in the serum. Previous studies reported that lower amino acid and glucose levels suppress mTOR activity, reducing protein synthesis but increasing autophagy and mitochondrial biogenesis, thereby improving metabolic outcomes [54,55]. Autophagy supports sperm maturation and motility by removing damaged components, whereas mitochondrial biogenesis ensures that sperm have the energy needed for movement [56]; This may underlie the beneficial effects of IF on spermatogenesis. In the testes, the most significant changes were observed in lipid metabolism, particularly in phospholipid metabolism and steroid biosynthesis, which are crucial for steroid hormone synthesis and spermatogenesis [43]. These tissue-specific metabolic shifts imply that IF induces systemic metabolic adaptation while also impacting testicular function by modulating lipid metabolism to preserve reproductive capability. Notably, both the serum and testicular samples exhibited metabolic pathway reprogramming, suggesting that IF may affect testicular metabolism through systemic signaling, thereby regulating endocrine function and male fertility.

To explore the relationship between the metabolic benefits of IF and testicular gene expression, we performed testicular transcriptomic analysis. Consistent with previous studies, the enrichment of biological processes related to cell adhesion, response to external stimuli, and response to bacteria suggested that a HFD induces inflammation and structural changes in the testes, potentially leading to tissue damage and an inflammatory state [57,58]. In contrast, the key enriched biological processes in the comparison between the IF and HFD groups highlighted the potential mechanisms of IF in the nutrient response, hormone regulation, insulin secretion, and reactions to external stimuli. These findings align with our metabolite analysis, suggesting that IF promotes spermatogenesis and improves testicular function by balancing hormone levels, enhancing glycolipid metabolism, and reducing oxidative stress. Specifically, IF downregulated the expression of genes related to lipid metabolism, inflammation, and immune responses, such as proopiomelanocortin (Pomc), phosphoenolpyruvate carboxykinase 1 (Pck1), leptin (Lep), erythroblast membrane-associated protein (Ermap), Wiskott-Aldrich syndrome protein (Was), BMX non-receptor tyrosine kinase (Bmx), protein tyrosine phosphatase receptor type C (Ptprc), and schlafen 1 (Slfn1), suggesting its potential for mitigating immune dysfunction and chronic inflammation induced by a HFD. Notably, Pomc, a regulator of satiety and energy balance, and Pck1 are involved in lipogenesis [59]. Lep, which encodes the proinflammatory adipokine leptin, can increase oxidative stress through the janus kinase-signal transducer and activator of transcription (JAK-STAT), AMP-activated protein kinase (AMPK), and mechanistic target of rapamycin (mTOR) pathways, decreasing sperm quality by reducing their concentration and viability [60]. Our study revealed that IF decreased Lep gene expression, suggesting that IF may alleviate leptin-induced inflammation, potentially improving the testicular microenvironment and sperm quality. Furthermore, the decrease in the expression of genes related to T cell activation (Ermap), antigen signaling (Was), and T cell and B cell activation (Ptprc and Slfn1) also reflects the anti-inflammatory effects of IF [61,62,63,64]. IF upregulated the expression of genes such as forkhead box A2 (Foxa2), adrenoceptor alpha 2A (Adra2a), and matrix metallopeptidase 7 (Mmp7), which are linked to peptide metabolism and the regulation of insulin and hormone secretion, indicating that IF contributes to maintaining glucose levels and metabolic health. Foxa2, a vital transcription factor, is essential for β cell glucose sensing and stability [65]. Foxa2 deficiency in mouse pancreatic cells under HFD conditions leads to increased obesity and reduced glucose uptake and glycolysis [66]. By enhancing Foxa2 expression, IF may reverse HFD-induced metabolic disturbances, providing energy for spermatogenesis and improving sperm quality and reproductive health.

Regarding the integrated metabolome analysis of serum and testis, IF was found to increase the levels of allantoin—a product of purine metabolism resulting from the oxidation of uric acid by the enzyme Uox [67]. Allantoin is known to activate imidazoline receptors, increase energy metabolism, and exert antioxidant and anti-inflammatory effects [68,69,70]. Previous studies demonstrate that a HFD significantly reduces hepatic Uox expression and allantoin levels, thereby decreasing serum concentrations and disrupting its natural rhythmic expression [71,72]. Furthermore, Uox-knockout mice display impaired glucose tolerance and predisposition to diabetes, hypertension, and dyslipidaemia, among other metabolic disorders [73]. In contrast, another study revealed that IF elevates hepatic Uox expression across multiple zeitgeber time (ZT) points compared to acute fasting, suggesting temporal regulation patterns in metabolic adaptation [74]. Consistent with its known effects, our results suggest that allantoin is negatively associated with inflammation, immune responses, steroid metabolism, and insulin resistance but positively influences energy homeostasis in the testis. These effects indicate that allantoin may play a key role in the metabolic and reproductive health benefits conferred by IF. Interestingly, owing to the gradual accumulation of genetic mutations during evolution, humans do not produce uricase. Thus, we hypothesize that IF in humans may, on the one hand, exert health benefits by altering the expression of upstream transcription factors, such as basic helix-loop-helix family member E40 (BHLEH40), which in turn regulate downstream metabolic pathways such as circadian rhythm, lipid metabolism, and protein stability (Supplementary Figure S3). On the other hand, while humans do not produce uricase per se, increasing evidence suggests that the gut microbiome is a key source of uricase, with anaerobic bacteria compensating for the loss of uricase genes [75]. Clinical studies have shown that IF enhances gut microbiota diversity, increasing the abundance of anaerobic bacteria such as *Roseburia*, *Clostridium* and *Bacteroides* [76,77,78]. This may be a mechanism through which IF influences uric acid metabolism and enhances the benefits of allantoin.

Our study indicates that IF may alleviate obesity-related reproductive dysfunctions by modulating specific genes and metabolites. While we demonstrated that IF combined with a high-fat diet during feeding windows (HFD-IF group) substantially attenuates its metabolic benefits and likely diminishes improvements in obesity-associated male infertility, the absence of a dedicated HFD-IF experimental group precludes definitive conclusions about its reproductive consequences. Future studies should incorporate this cohort to directly assess its effects on fertility parameters and elucidate mechanistic links between dietary composition and IF efficacy. It is also crucial to explore the molecular mechanism of allantoin and its potential as a therapeutic target. Translating these findings to humans requires caution due to species-specific differences in fasting physiology, such as hormonal dynamics and spermatogenesis kinetics. Clinical trials are needed to evaluate IF’s effects on male fertility in populations with obesity, optimize dietary protocols during feeding windows, and validate biomarkers like allantoin in human biofluids. Nevertheless, our study provides multilevel molecular insights into obesity-induced male infertility and IF’s therapeutic potential.

## 5. Conclusions

In this study, for the first time, we demonstrated the effectiveness of IF in alleviating male infertility induced by obesity. By integrating testis metabolome and transcriptome analyses, we found that IF can regulate purine metabolism, modulate glycolipid metabolism, reduce immune disturbances, and improve energy homeostasis. Additionally, our multidimensional analysis highlighted the role of increased levels of allantoin in mediating the beneficial effects of IF on obese mice. Overall, we demonstrated the potential of IF in enhancing male fertility affected by obesity and provided a foundation for the use of IF as a lifestyle intervention for obesity-related infertility, paving the way for future research on the application of IF in humans. Future studies should validate these targets in dedicated preclinical models and accelerate translation through controlled human trials addressing species-specific physiological adaptations.

## Data Availability

The datasets used and analyzed during the current study are available from the corresponding author on reasonable request. The data are not publicly available due to ongoing experiments and partner-related considerations.

## Supplementary Materials

The following supporting information can be downloaded at: https://www.mdpi.com/article/10.3390/nu17061029/s1, Figure S1: Effects of high-fat-diet and intermittent fasting on testis metabonomic profiling; Figure S2: Effects of high-fat-diet and intermittent fasting on serum metabonomic profiling; Figure S3: Liver Uox IHC; Figure S4: Prediction of intermittent fasting effects on human UOX gene transcription factors and target gene enrichment; Table S1: The overlapped metabolites in the testis of the two compared groups; Table S2: The overlapped metabolites in the serum of the two compared groups; Table S3. The overlapped genes in the testis of the two compared groups.
