# Supplementary material for: Integrated Multiomics Analyses Reveal Molecular Insights into How Intermittent Fasting Ameliorates Obesity and Increases Fertility in Male Mice
Source: Nutrients. 2025 Mar 14;17(6):1029. doi: 10.3390/nu17061029 (PMC11945891; doi:10.3390/nu17061029)
Supplement: Supplementary file 1 [file nutrients-17-01029-s001.zip › nutrients-3515585-supplementary.pdf]

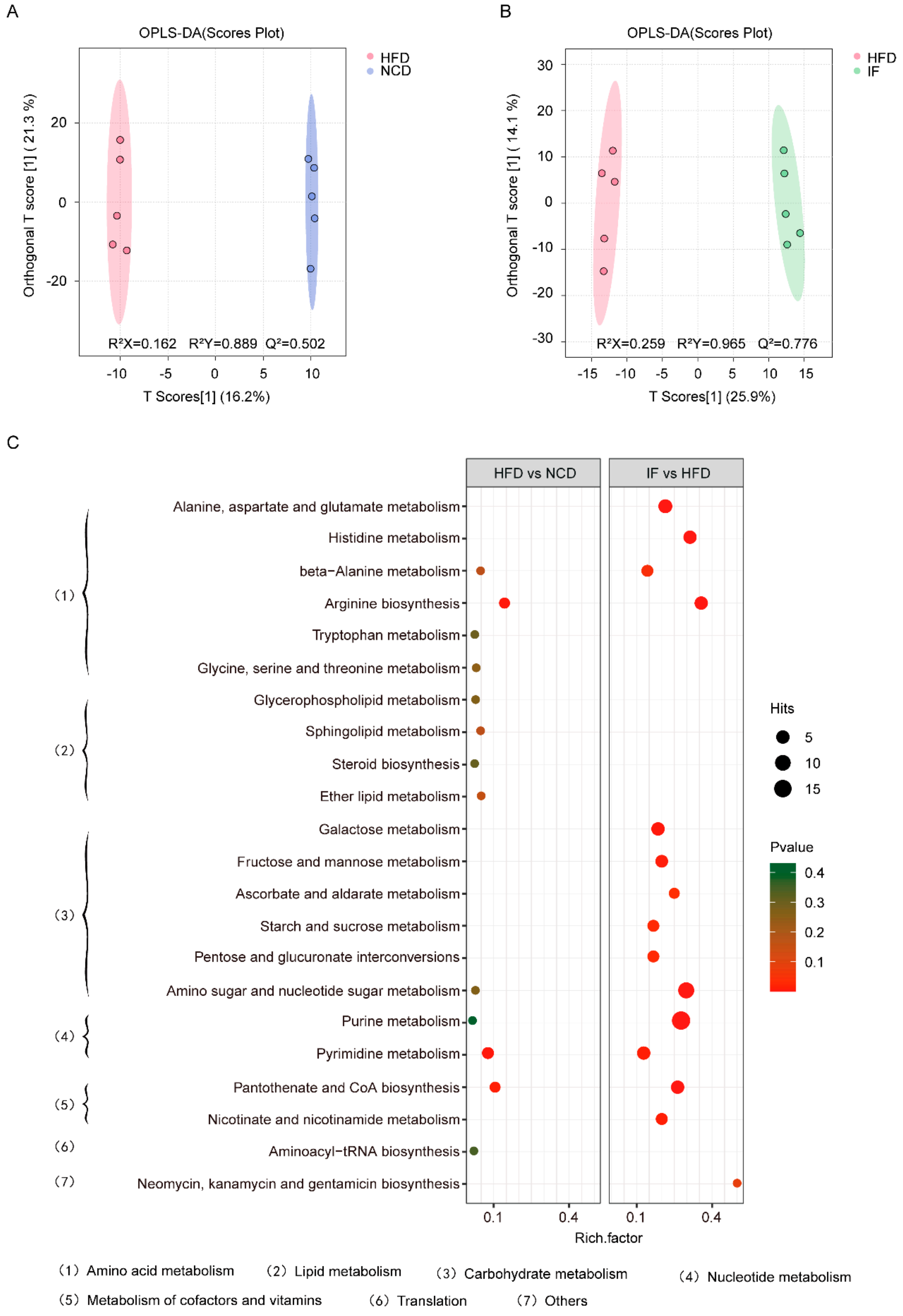

Figure S1 Effects of high-fat-diet and intermittent fasting on testis metabonomic profiling. (A) Orthogonal partial least square discriminant analysis (OPLS-DA) score plots of HFD vs NCD and (B) IF vs HFD. (C) Bubble diagram of enriched KEGG pathways. The horizontal coordinate is the extent to which the pathway is affected. Size of dots represents number of metabolites in each KEGG pathway. The P-values calculated by the enrichment analysis are described in terms of their color intensity.

Table S1 The overlapped metabolites in the testis of the two compared groups.

Class	Metabolite	HFD vs NCD			IF vs HFD		
		Log2FC	P-value	VIP	Log2FC	P-value	VIP
Lipids and lipid-like molecules	Glycerophosphoethanolamine	0.2208	0.0092	1.9791	-0.2773	0.0009	1.9204
	Glycerophosphocholine	0.1941	0.0185	1.9255	-0.2703	0.0079	1.7220
	Desmosterol	0.5847	0.0148	1.9517	-0.6368	0.0081	1.7536
	PC(16:0/20:4)	0.1309	0.0143	1.9364	-0.1494	0.0331	1.4771
	1-Palmitoyl-2-hydroxy-sn-glycero-3-phospho-(1'-rac-glycerol)	0.3087	0.0050	2.1012	-0.2566	0.0417	1.4427
	PC(44:8)	0.3843	0.0077	1.9988	-0.3559	0.0033	1.7985
	Oxononanoylcarnitine	1.9296	0.0008	2.2320	-0.9345	0.0147	1.5364
	Decatrienoylcarnitine	-1.5886	0.0027	2.1482	1.2372	0.0088	1.6639
	LPC(19:0)	-1.4045	0.0027	1.7858	1.3688	0.0049	1.4994
	1,2-Dipalmitoyl-sn-glycero-O-ethyl-3-phosphatidylcholine cation	0.6035	0.0367	1.7625	-0.6947	0.0295	1.4402
	Umbelliferyl arachidonate	0.3995	0.0297	1.8240	-0.3900	0.0227	1.5756
Organic acids	Phenylsulfate	-2.9479	0.0276	1.9751	2.9760	0.0145	1.7123
	3-Ureidopropionic acid	1.0715	0.0000	2.3884	-0.8959	0.0001	2.0367
	N-Acetylglutamic acid	0.4312	0.0012	2.2258	-0.4605	0.0017	1.8405
	Gly-His	0.2808	0.0331	1.6488	-0.3002	0.0035	1.8051
Organoheterocyclic compounds	Allantoin	-0.4903	0.0055	2.1361	0.7468	0.0054	1.9854
	3-Pyridylacetic acid	-3.9565	0.0011	2.4897	3.8180	0.0001	2.1213
	Dehydro-L-(+)-ascorbic acid dimer	0.3051	0.0082	1.9832	-0.3594	0.0107	1.6629

	Ascorbic acid	0.9652	0.0434	1.7892	-1.3530	0.0281	1.5989
Benzenoids	3-Aminobenzoic acid	-3.9565	0.0011	2.4897	3.8180	0.0001	2.1213
	2,4-Dimethoxybenzoic acid	0.5371	0.0288	1.8679	-0.5864	0.0209	1.6562
Polyketides	Hydroxyphenyllactic acid	0.5371	0.0288	1.8679	-0.5864	0.0209	1.6562
Alkaloids	Trigonelline	-3.9565	0.0011	2.4897	3.8180	0.0001	2.1213
Organic nitrogen compounds	Trimethylamine N-oxide	-0.7994	0.0023	2.2821	0.7495	0.0004	2.0072
Others	1-(5'-Phosphoribosyl)-5-amino-4-imidazolecarboxamide	0.1660	0.0141	1.9376	-0.2304	0.0457	1.4450
	Propanoyl phosphate	0.3637	0.0185	1.7995	-0.2930	0.0021	1.8641

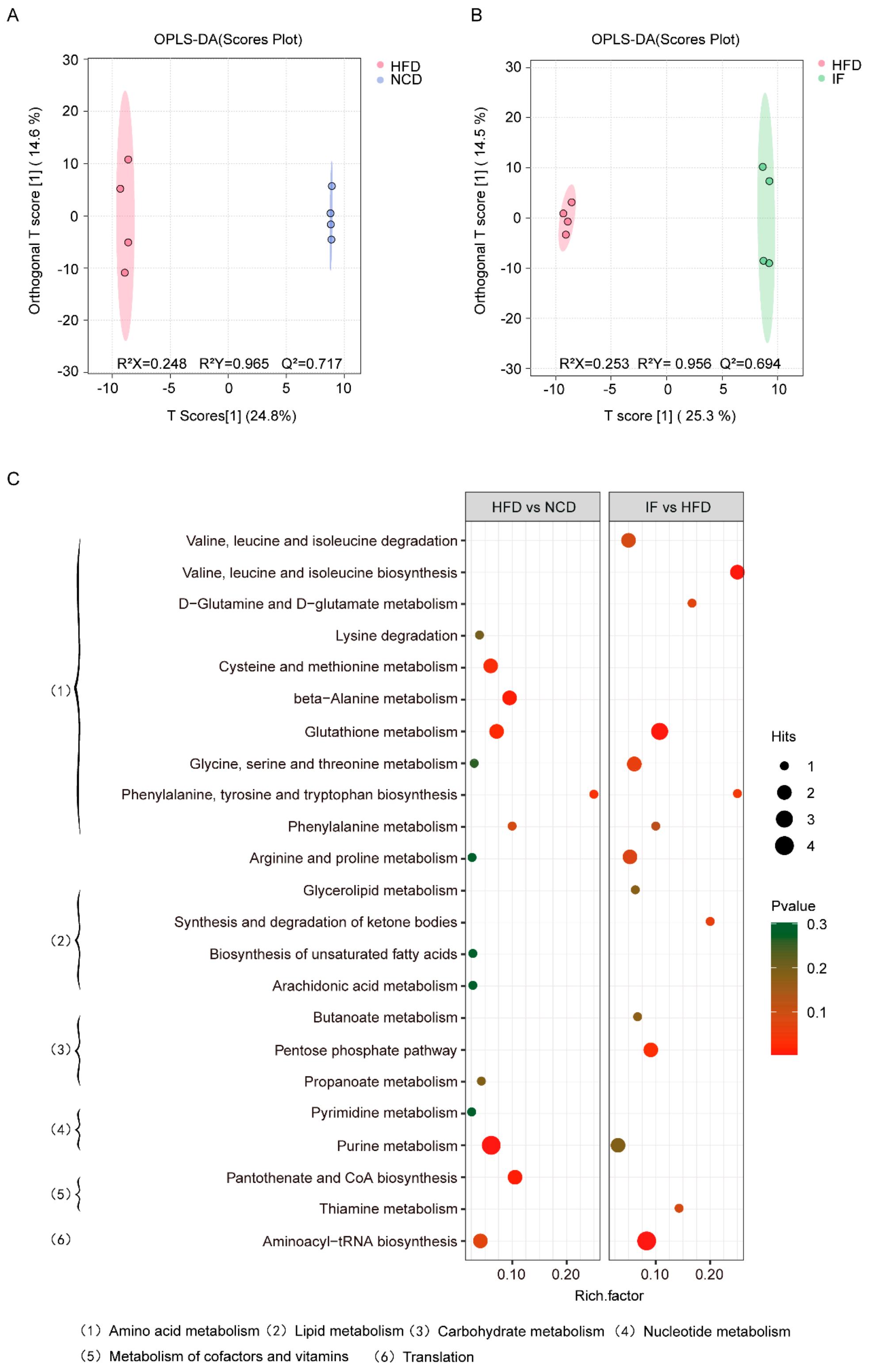

Figure S2 Effects of high-fat-diet and intermittent fasting on serum metabonomic profiling. (A) Orthogonal partial least square discriminant analysis (OPLS-DA) score plots of HFD vs NCD and (B) IF vs HFD. (C) Bubble diagram of enriched KEGG pathways. The horizontal coordinate is the extent to which the pathway is affected. Size of dots represents number of metabolites in each KEGG pathway. The P-values calculated by the enrichment analysis are described in terms of their color intensity.

Table S2 The overlapped metabolites in the serum of the two compared groups.

Class	Metabolite	HFD vs NCD			IF vs HFD		
		Log2FC	P-value	VIP	Log2FC	P-value	VIP
Organic acids	Creatinine	0.2349	0.0461	1.4137	-0.3825	0.0191	1.5611
	Diaminopimelate	0.2104	0.0124	1.6554	-0.3557	0.0027	1.7738
	Glutathione	1.4636	0.0002	1.9050	-1.9300	0.0244	1.5530
	Cymethion	0.4728	0.0045	1.7593	-0.3849	0.0071	1.6989
	Tetrahydrofolate	-0.1849	0.0390	1.4842	0.4767	0.0298	1.4921
	Methionine	0.4728	0.0045	1.7593	-0.3849	0.0071	1.6989
	Phenylalanine	-0.1849	0.0390	1.4842	0.4767	0.0298	1.4921
	DAPA	0.2104	0.0124	1.6554	-0.3557	0.0027	1.7738
Lipids and lipid-like molecules	LPC 14:0	-1.0953	0.0004	1.8919	0.7830	0.0073	1.6871
	Perillic acid	0.1722	0.0126	1.6392	-0.2096	0.0036	1.7599
	1-Myristoyl-GPC	-1.0953	0.0004	1.8919	0.7830	0.0073	1.6871
	Stearoyl-L-Carnitine	0.8085	0.0225	1.5617	-0.9774	0.0148	1.6332
Organoheterocyclic compounds	Allantoin	-0.4153	0.0164	1.6172	0.5784	0.0452	1.4123
Organic nitrogen compounds	Spermidine	-0.6602	0.0389	1.4716	0.4115	0.0182	1.5810

Table S3 The overlapped genes in the testis of the two compared groups.

Symbol/Ensembl ID	HFD vs NCD		IF vs HFD	
	Log2FC	P-value	Log2FC	P-value
Zfp750	-1.62661	0.004824	1.461965	0.013337
Wnt11	-1.99029	0.001503	1.764735	0.008629
Was	2.867435	6.00E-05	-1.26224	0.020175
Ugt2b34	2.059424	0.016245	-2.12251	0.012622
Traf3ip3	2.076055	0.045087	-2.07605	0.045646
Teddm1a	2.588156	0.006517	-2.27238	0.030418
Slfn1	1.876977	0.047398	-2.53215	0.014259
Slc36a3os	2.617056	0.005618	-2.61706	0.005428
Slc19a3	2.286578	0.044107	-2.38521	0.025585
Ptprc	1.048828	0.001749	-1.15332	0.009903
Prdm8	-5.73319	2.12E-19	4.372539	3.75E-05
Pomc	1.899412	0.009551	-2.29687	0.007505
Pde2a	-5.1025	4.91E-07	5.159925	9.06E-07
Pck1	1.837394	0.000685	-1.336	0.003013
Pced1b	-1.53492	1.38E-06	1.766984	0.002191
Pcdhgc4	4.040211	0.000846	-3.79827	0.002422
Olfr736	2.110818	0.042459	-2.11082	0.042882
Myl7	-2.32723	0.011443	3.050705	0.000242
Mybphl	2.110818	0.042459	-2.11082	0.042882
Muc6	1.327125	0.011316	-1.0328	0.035011
mt-Ts2	-1.7354	0.01539	3.414478	2.78E-07
Ms4a6d	1.315893	7.13E-05	-1.39544	1.50E-05
Ms4a6c	1.014182	0.012579	-1.36313	2.16E-05
Mrgpre	-2.08922	0.043116	1.761004	0.036578
Mmp7	-4.78598	7.90E-05	2.991015	0.000506
Mab21l3	-2.69867	0.002395	2.630232	0.005219
Lgals12	-1.76569	0.046288	1.952736	0.019815
Lep	1.549556	0.012421	-1.43925	0.010812
Kcnn2	-1.0662	0.042349	1.242386	0.014367
Igkv6-20	2.301348	6.50E-08	-2.30135	1.97E-08
Ighv4-1	2.252704	7.91E-08	-2.2527	2.42E-08
Ighv15-2	2.417153	0.033339	-3.28079	0.000134
Hrh4	1.001621	0.012415	-1.00694	0.012826
Gpr84	-1.68273	0.049568	3.04987	0.000455
Gm7965	2.220866	0.02788	-2.22087	0.027885
Gm7199	-2.03511	0.047587	2.053324	0.042388
Gm5424	-1.28871	1.28E-05	1.335108	7.76E-06
Gm4613	-4.2729	0.032393	2.162194	1.20E-06
Gm45837	1.490071	0.003606	-1.86082	0.000174

Gm45799	3.493808	3.96E-06	-1.41781	0.007032
Gm45493	1.869682	0.044163	-1.34988	0.044089
Gm43339	1.386773	0.019473	-1.66619	0.002447
Gm43225	2.303784	0.03484	-2.94828	0.001063
Gm37534	1.32632	0.042457	-1.74821	0.008344
Gm37518	1.80735	0.044479	-2.82428	0.003546
Gm37108	1.773938	0.038807	-1.77394	0.039932
Gm33571	1.277275	0.046197	-1.27727	0.047825
Gm29683	1.069301	0.032638	-1.61714	0.002119
Gm28178	2.169055	0.023996	-2.16905	0.024037
Gm20547	3.556379	0.023784	-2.20124	3.26E-05
Gm20541	2.042249	0.012703	-1.86926	0.031828
Gm20431	3.383047	1.88E-09	-3.38305	1.54E-10
Gm16876	1.98289	0.031346	-3.23154	0.000193
Gm15592	3.246553	0.000207	-3.24655	0.000182
Gm13147	1.19962	0.019816	-1.11847	0.03945
Gm12018	1.74973	0.007405	-1.16442	0.042505
Gm11586	2.24124	0.026965	-1.57157	0.009739
Gm10827	-2.17062	0.00308	1.528497	0.015917
Gm10774	1.175698	0.024983	-1.21914	0.022865
Gm10252	-2.20407	0.023554	1.740976	0.039461
Gimap8	1.536473	0.001693	-1.61114	0.002185
Gbp9	1.076679	0.004426	-1.73149	7.09E-05
Foxa2	-2.28566	0.030644	2.115562	0.047801
Ermap	1.264174	0.024699	-1.04876	0.049846
Dleu7	3.069603	0.001375	-1.31405	0.033943
Ddc	3.051554	0.001702	-1.88755	0.036327
D430018E03Rik	1.306377	0.008364	-1.46586	0.007232
Clca3b	2.322877	0.020431	-2.32288	0.020339
Ces5a	-1.73854	0.025402	-1.01177	0.001262
Ceacam10	-2.01801	0.032227	-1.05579	0.025495
Btnl2	-1.97334	0.017156	1.784587	0.040493
Bmx	1.329123	0.018923	-1.56774	0.007813
B430203G13Rik	2.768157	0.002868	-2.76816	0.002719
Adra2a	-2.55484	0.006063	1.911537	0.025552
Acss2os	-2.2914	0.033913	2.275123	0.03712
Acbd7	-2.6252	0.029039	-1.95729	0.024717
AC192334.3	2.521305	0.005347	-2.04446	0.048831
AC158232.1	2.003914	0.020838	-3.48899	4.24E-05
AC156273.2	1.900421	0.031492	-2.19704	0.012569
AC155909.1	2.212415	0.016861	-2.22619	0.001886
AC153501.1	1.335099	0.018582	-2.37901	0.004502
AC099934.1	1.381179	0.005951	-1.42462	0.005462
AC079644.12	-2.88099	0.001304	2.17584	0.027848

A530041M06Rik	1.877184	0.011421	-2.66813	0.018744
A230065N10Rik	-2.3634	0.015452	1.841322	0.022608
9230102O04Rik	1.405741	0.001063	-2.10031	2.23E-06
6430590A07Rik	1.238588	0.018456	-1.07277	0.022628
4932441J04Rik	-1.04835	0.0379	1.178261	0.015335
4930590L20Rik	1.955806	0.0184	-1.95581	0.018843
4930515G16Rik	1.511938	0.016941	-2.24508	0.005512
4930500L23Rik	1.928969	0.023043	-1.92897	0.023401
4930426D05Rik	-1.04818	0.017304	1.05841	0.016102
2900092O11Rik	2.225837	0.029419	-2.20034	0.029049
2810455O05Rik	1.578299	0.006447	-1.0546	0.032826
1700049E15Rik	2.864008	0.000368	-2.86401	0.000339
1700028M03Rik	1.051798	0.037211	-1.03797	0.042213
ENSMUSG00000116802	1.053821	0.00454	-1.60816	0.000941
ENSMUSG00000116954	1.150827	0.032699	-1.09784	0.00786
ENSMUSG00000117641	1.818584	0.045333	-1.81858	0.046611
ENSMUSG00000117992	1.544735	0.011666	-1.19697	0.035192
ENSMUSG00000116959	2.057508	0.039448	-2.05751	0.039679
ENSMUSG00000118109	1.703597	0.017552	-1.29151	0.018247
ENSMUSG00000116249	1.198791	0.044398	-1.34904	0.023878
ENSMUSG00000117063	2.469961	3.21E-08	-2.46996	9.68E-09
ENSMUSG00000117885	2.520649	0.007013	-2.52065	0.006787

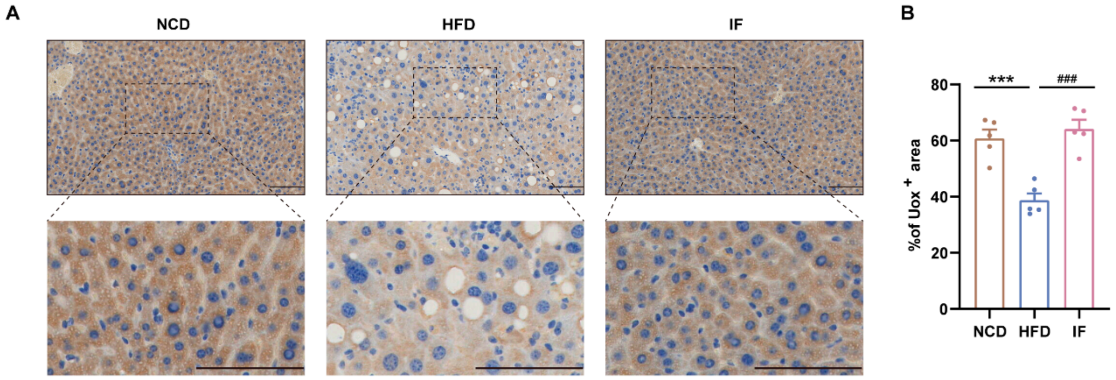

Figure S3 Liver Uox IHC. (A) Representative Uox IHC staining images. (B) Quantification of Uox-positive stained areas (scale bar, 100μm) (n = 5 mice per group). Results shown as mean ± SEM. One-way ANOVA was used for comparing the differences among indicated groups (***P < 0.001 vs NCD; ###P < 0.001 vs HFD)

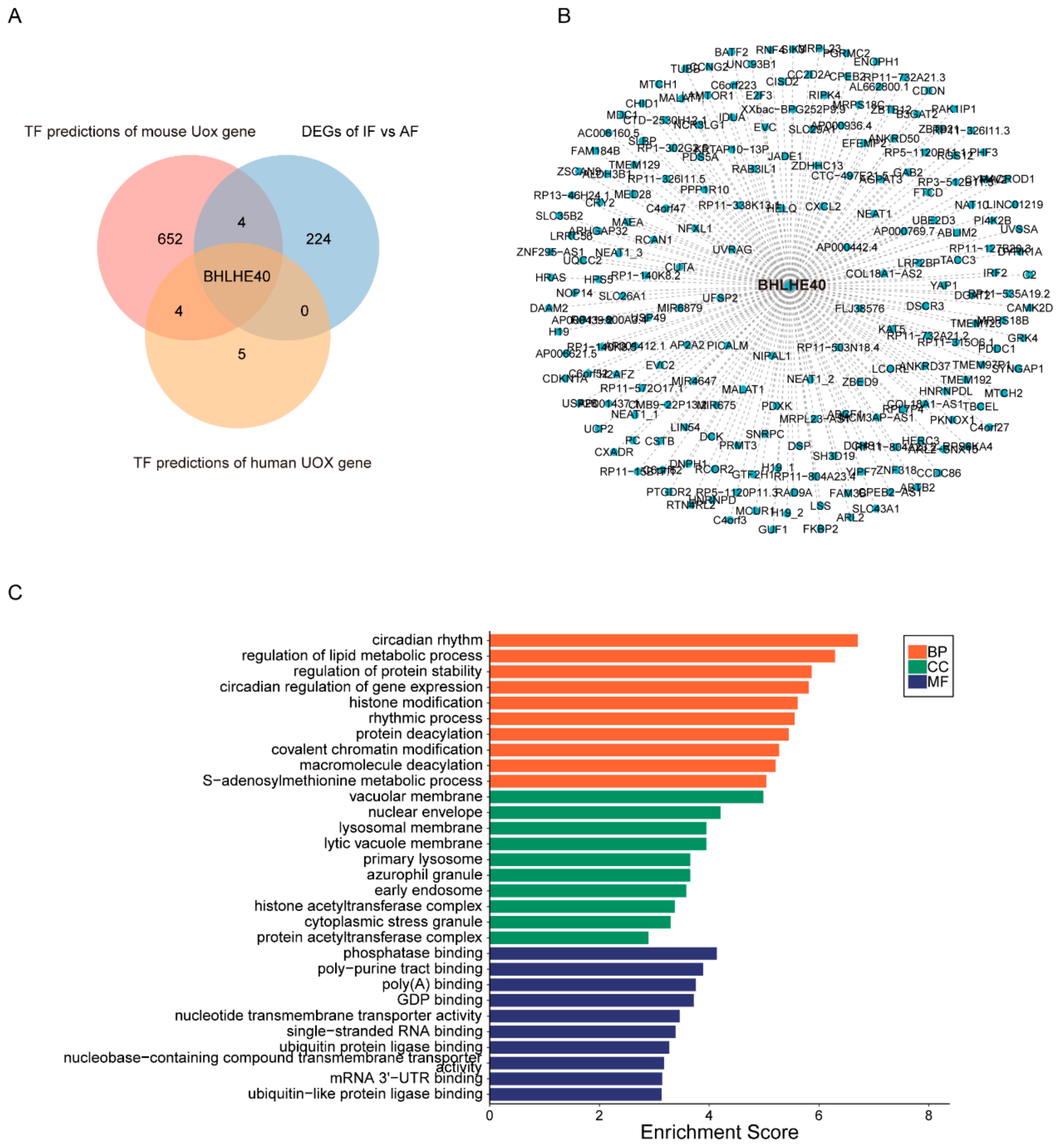

Figure S4 Prediction of intermittent fasting effects on human UOX gene transcription factors and target gene enrichment. (A) Venn plot showing the overlap genes in the three databases.TF predictions of mouse Uox gene were obtained from the JASPAR CORE vertebrates collection (2022). TF predictions of human Uox gene were obtained from ENCODE Transcription Factor Targets. DEGs of IF vs AF were obtained from GSE154797. (B) Top 200 BHLHE40-target genes predict from hTFtarget databetes. (C) GO Results of BHLHE40 target genes predict from hTFtarget databetes.
